# Participatory ergonomic intervention versus strength training on chronic pain and work disability in slaughterhouse workers: study protocol for a single-blind, randomized controlled trial

**DOI:** 10.1186/1471-2474-14-67

**Published:** 2013-02-21

**Authors:** Emil Sundstrup, Markus D Jakobsen, Christoffer H Andersen, Kenneth Jay, Roger Persson, Per Aagaard, Lars L Andersen

**Affiliations:** 1 National Research Centre for the Working Environment, Lersø Parkalle 105, Copenhagen, Denmark; 2 Institute for Sports Science and Clinical Biomechanics, University of Southern Denmark, Odense, Denmark

**Keywords:** Musculoskeletal disorders, Occupational health, Shoulder pain, Tennis elbow, Repetitive and forceful movements

## Abstract

**Background:**

The prevalence of musculoskeletal pain in the shoulder, arm and hand is high among slaughterhouse workers, allegedly due to the highly repetitive and forceful exposure of these body regions during work. Work disability is a common consequence of these pains. Lowering the physical exposure through ergonomics intervention is the traditional strategy to reduce the workload. An alternative strategy could be to increase physical capacity of the worker through strength training. This study investigates the effect of two contrasting interventions, participatory ergonomics versus strength training on pain and work disability in slaughterhouse workers with chronic pain.

**Methods/design:**

66 slaughterhouse workers were allocated to 10 weeks of (1) strength training of the shoulder, arm and hand muscles for 3 x 10 minutes per week, or (2) participatory ergonomics involving counseling on workstation adjustment and optimal use of work tools (~usual care control group). Inclusion criteria were (1) working at a slaughterhouse for at least 30 hours per week, (2) pain intensity in the shoulder, elbow/forearm, or hand/wrist of at least 3 on a 0–10 VAS scale during the last three months, (3) pain lasting for more than 3 months, (4) frequent pain (at least 3 days per week) (5) at least moderate work disability, (6) no strength training during the last year, (7) no ergonomics instruction during the last year.

Perceived pain intensity (VAS scale 0–10) of the shoulder, elbow/forearm and hand/wrist (primary outcome) and Disability of the Arm, Shoulder and Hand (Work module, DASH questionnaire) were measured at baseline and 10-week follow-up. Further, total muscle tenderness score and muscle function were assessed during clinical examination at baseline and follow-up.

**Discussion:**

This RCT study will provide experimental evidence of the effectiveness of contrasting work-site interventions aiming at reducing chronic pain and work disability among employees engaged in repetitive and forceful work.

**Trial registration:**

ClinicalTrials.gov:NCT01671267

## Background

Musculoskeletal disorders represent the most common occupational diseases in the European Union [1]. Besides the direct effect on employee health and work disability, work-related musculoskeletal disorders impose a major socioeconomic burden due to extensive use of health care services, sickness absence, disability pension and loss of productivity [2-6]. The prevalence of work-related musculoskeletal disorders, especially in the shoulder, neck and upper extremity is higher in occupations involving a high rate of repetitive movements compared with less repetitive job settings [7-9]. In 2005 about 23% of European workers reported that their work negatively affected their health in the form of significant pain in the shoulder, neck, and/or upper/lower limbs [1]. In a Danish survey approximately one third of the general workforce reported moderate to severe neck/shoulder pain [3]. Thus worksite interventions to effectively prevent and rehabilitate musculoskeletal pain in these anatomical regions seem highly needed.

Slaughtering and meat processing operations involves a high degree of repetitive and forceful upper limb movements and implies an elevated risk of work-related musculoskeletal disorders [10,11]. The rate of nonfatal occupational injuries and illnesses for workers engaged in animal slaughtering is more than twice as high as the US-national average, and the number of cases with days away from work, job transfer, or restriction are almost three times the national average [12]. Especially the prevalence of musculoskeletal disorders in the shoulder, arm and hand is high among slaughterhouse workers, allegedly due to high loading intensities and cyclic repetitive actions of these body regions during work [7-9,13]. The increased prevalence of musculoskeletal disorders is associated with several work-related risk factors including highly repetitive and forceful exertion, lack of sufficient recovery and awkward postures [7,14]. Furthermore, for hygienic reasons slaughterhouse temperature is often low, leading to increasing cutting resistance of the meat and thereby increased risk of developing pain in the arm, shoulder and hand [15-17]. Temporary work disability is a common consequence of the above scenario, manifested by pain in the arm, shoulder and hand, with psychosocial factors related to the job and work environment playing an additional role in the development of work-related musculoskeletal pain [7,18,19]. Monotonous work, limited job control, poor self-efficacy and low social support at work have been associated with various musculoskeletal disorders [7].

The recent implementation of mechanically based production systems in the meat cutting industry seems to reduce the variation in biomechanical exposure. The preferable long work-cycles combined with a variety of movements is currently being replaced by machine directed line-production systems with shorter work-cycles and a higher degree of repetitive and monotonous work tasks [20]. However, not all work tasks can be automatized, leaving a great deal of cutting and lifting tasks to be carried out manually, often using piece-rate salary systems [21]. Common for all these manual operations is the repetitive and forceful arm, shoulder and hand motions and inclined/reclined body postures, which has been associated with nonspecific muscle-tendon pain and related to musculoskeletal disorders such as shoulder tendinitis, epicondylitis, hand and wrist tendinitis and carpal tunnel syndrome (CTS) [7,13,22]. Also, not only the dominant side is prone for developing musculoskeletal disorders in slaughterhouse workers. Falck and Aarnio [23] reported an elevated flexor carpi radialis muscle activity in the left (assisting) vs. right (knife handling) side, and a clustering of left-sided CTS has previously been observed among slaughter workers [24]. Thus, motions such as tearing and holding with the assisting (non-dominant) hand may increase exposure to the non-dominant side as well.

Only few quantitative studies have assessed musculoskeletal disorders in slaughterhouse workers, while mainly focusing on ergonomic exposure evaluated by biomechanical analysis and risk factors associated with specific meat cutting operations. Madeleine & Madsen [25] reported a more stable motor strategy, including shorter work cycle duration, smaller range of motion and less movement variability and higher complexity during a de-boning task in experienced workers compared with less experienced workers. Further, pain and discomfort in the shoulder/neck region have been associated with changes in motor activity patterns, in particular characterized by decreased motor variability [25,26]. Muscle pain seems to influence shoulder and neck posture, while also leading to altered spinal loading patterns during specific lifting tasks [27,28].

Sharpness of blades used in meat cutting operations has been extensively studied, demonstrating associations with reduced force exposure in the upper extremity [29,30]. MvGorry and co-workers [31] showed that tool and workstation modifications scaled to the individual meat cutter could minimize wrist deviation, which is ergonomically desirable, and improve upper extremity posture.

Lowering the physical exposure through participatory ergonomic interventions may represent a strategy to reduce musculoskeletal loading intensity and/or rehabilitate musculoskeletal pain. A review by Rvilis et al. [32] found partial to moderate evidence that participatory ergonomic interventions are effective in improving different health outcomes. The main reason for not finding full evidential support was due to the low number of methodologically sound studies available in the literature. In opposition, pooled data obtained in a subgroup of employees with musculoskeletal disorders indicated that workplace interventions may be effective in reducing sickness absence, but not effective in improving general health outcomes [33]. Despite lack of scientific evidence, ergonomic training and education seems to be the general worksite approach on the prevention and treatment of musculoskeletal disorders.

An alternative strategy to reduce or prevent work-related musculoskeletal pain may be achieved by increasing the workers physical capacity through strength training interventions. Previous studies from our research group have shown promising and effective reductions in neck/shoulder/arm pain in response to 10–20 weeks of strength training using kettlebells [34,35], elastic rubber bands [36,37] or free weight exercises [19,38,39] in office workers and laboratory technicians. However, office workers and laboratory technicians have vastly different working conditions than slaughterhouse workers, and our previous findings may therefore not be directly transferable to other occupational groups. Nevertheless, from a theoretical point of view, increasing physical capacity by means of on-site progressive strength training of the shoulder, arm and hand muscles may provide an alternative way of reducing chronic pain and work disability in slaughterhouse workers. On the other hand, slaughterhouse workers are exposed to highly repetitive high-force work tasks that may hinder adequate recovery between subsequent strength training sessions. Therefore, relevant grounds exist to investigate whether strength training is a relevant and feasible intervention modality compared with a more traditional participatory ergonomics approach in slaughterhouse workers.

The aim of this study was to investigate the effect of two contrasting interventions, i.e. load reduction (participatory ergonomic intervention) versus increased physical capacity (strength training) on musculoskeletal pain and work disability in slaughterhouse workers with chronic musculoskeletal pain of the shoulder, elbow/forearm, and/or hand/wrist.

## Methods/design

### Trial design

A two-armed parallel-group, single-blind, randomized controlled trial with allocation concealment is currently conducted among slaughterhouse workers in Denmark. The participants were allocated to a 10 week intervention period and paralleled assigned to receive either strength training or ergonomics at the worksite. The study duration is August 2012 to January 2013.

### Ethics

The study was approved by The Danish National Committee on Biomedical Research Ethics (The local ethical committee of Frederiksberg and Copenhagen; H-3-2010-062) as part of the research program *“Implementation of physical exercise at the workplace (IRMA)”*. The trial *“Implementation of Physical Exercise at the Workplace (IRMA06) - Slaughterhouse Workers”* was registered in ClinicalTrials.gov (NCT01671267") prior to enrolment of participants.

### Participants

66 slaughterhouse workers were recruited from 2 slaughterhouses in Denmark. All participants were informed about the purpose and content of the project and gave their written informed consent to participate in the study. All experimental conditions conformed to The Declaration of Helsinki.

### Recruitment

The recruitment was two-phased and consisted of a brief screening questionnaire in June 2012 followed by a clinical examination and questionnaire in Aug-Sept 2012.

Firstly, in June 2012 a screening questionnaire was administered to 645 slaughterhouse workers (aged 18–67 years) in two slaughterhouses in Denmark. In total 595 replied to the questionnaire of which 410 were interested to participate in the research project. The initial inclusion criteria based on the screening questionnaire were: (1) working at a slaughterhouse for at least 30 hours a week, (2) pain intensity in the shoulder, elbow/forearm, or hand/wrist of at least 3 on a 0–10 scale during the last 3 months, (3) at least *“some”* work disability scoring on a five-point scale: *“not at all”*, *“a little”, “some”, “much”* to “*very much”* using the question *“During the last 3 months, did you have any difficulty performing your work due to pain in the shoulder, arm or hand”*, (4) no strength training during the last year, (5) no ergonomics instruction during the last year. Of the 410 interested respondents, 145 met the above criteria and were invited for a clinical examination in Aug-Sept 2012. Characteristics of slaughterhouse workers who accepted and declined participation are illustrated in Table 1.

**Table 1:** Characteristics of slaughterhouse workers who accepted (Yes) and declined (No) participation in the intervention

	**Yes**	**No**	**p - value**
N	410	185	ns
Age (years)	43 (9)	44 (10)	ns
Height (cm)	177 (9)	176 (8)	ns
Weight (kg)	83 (17)	84 (16)	ns
BMI (kg∙m^-2^)	27 (5)	27 (5)	ns
Shoulder pain intensity during the last 3 months (scale 0–10)	4 (2.8)	2.9 (2.7)	<.0001
Elbow pain intensity during the last 3 months (scale 0–10)	2.8 (2.8)	1.9 (2.4)	<.0001
Forearm pain intensity during the last 3 months (scale 0–10)	2.3 (2.6)	1.4 (2.1)	<.0001
Hand/Wrist pain intensity during the last 3 months (scale 0–10)	3.6 (2.9)	2.7 (2.9)	<.001

Values are means (SD). P-values denotes levels between those accepting and declining participation. ns = not significant.

A total of 135 employees presented for the baseline clinical examination. Exclusion criteria were (1) hypertension (Systolic BP > 160, diastolic BP > 100), (2) a medical history of cardiovascular diseases (e.g. chest pain during physical exercise, heart failure, myocardial infarction and stroke), (3) recent traumatic or severe injury to the neck, shoulder, arm or hand regions, or (4) pregnancy. Furthermore, at the day of the clinical examination participants filled in another questionnaire with the following inclusion criteria: (1) pain intensity in the shoulder, elbow/forearm, or hand/wrist of at least 3 on a 0–10 VAS scale during the last week, (2) pain should have lasted more than 3 months, (3) frequency of pain of at least 3 days per week during the last week.

**Figure 1:**
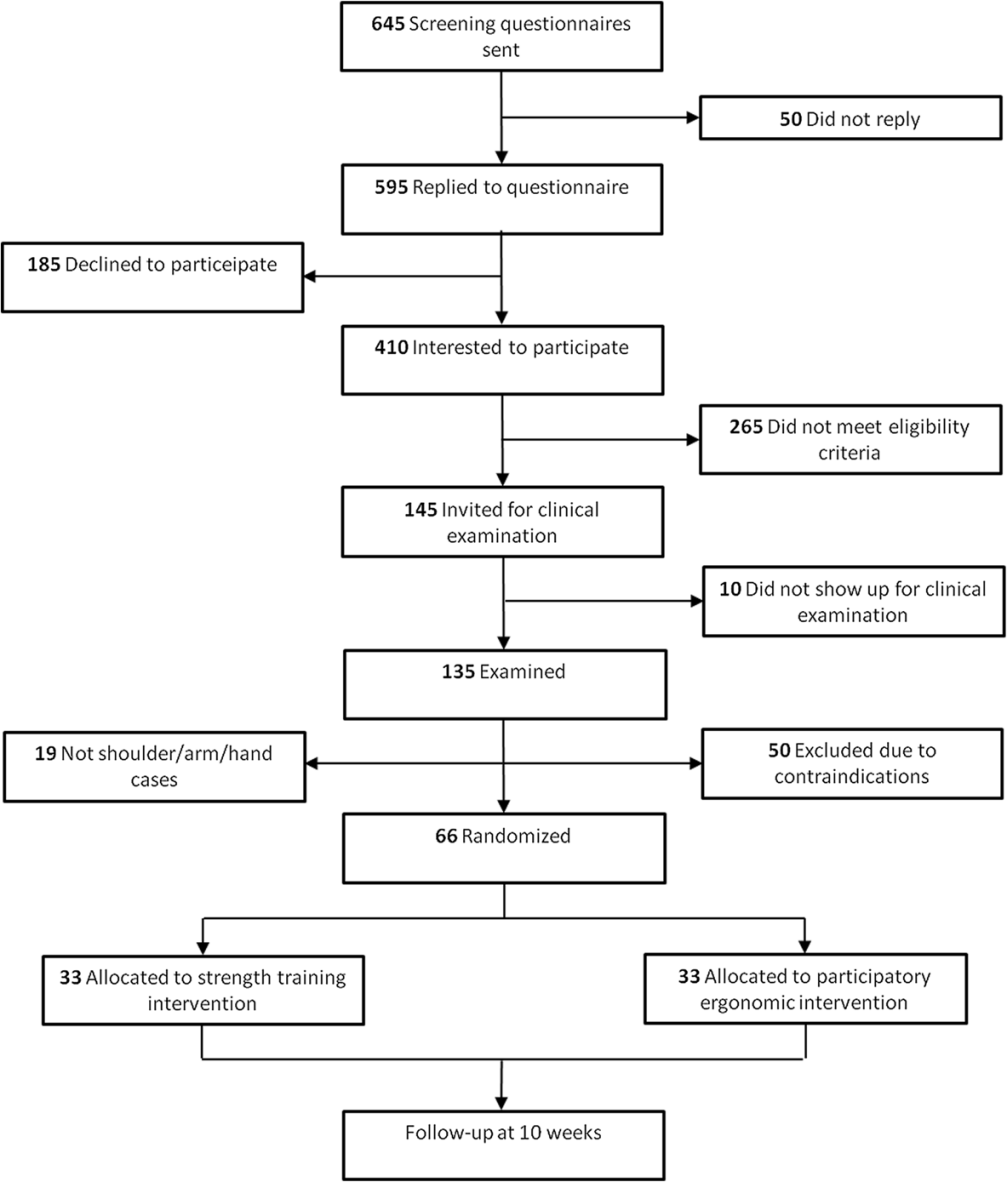
Flow-chart.

During the clinical examination and associated questionnaire, 69 workers were excluded due to contraindications: 19 showed symptoms of carpal tunnel syndrome, 4 had blood pressure above 160/100 mmHg, 1 had a serious cardiovascular disease, and 19 did not meet the pain inclusion criteria. Furthermore, 26 were excluded because they did not speak or understand Danish to a degree that they were able to fill in the questionnaire. The flow of participants is illustrated in Figure 1.

### Randomization

On the basis of the clinical examination and associated questionnaire we randomly allocated the 66 eligible participants, using a computer-generated random numbers table (SAS), to either strength training intervention or ergonomic counseling. Gender and worksite (2 slaughterhouses) were used as stratification variables. Subsequently, participants were informed by letter about group allocation. At the follow-up physical examination and questionnaire in Dec 2012-Jan 2013, all examiners were blinded, and participants instructed not to reveal their particular intervention. Baseline characteristics and pain score of employees randomized into the two intervention groups are illustrated in Table 2.

**Table 2:** Characteristics of the two intervention groups

	**Participatory ergonomics**	**Strength training**
N	33	33
Number of men/women	26/7	25/8
Age (years)	43 (9)	48* (9)
Height (cm)	177 (9)	174 (10)
Weight (kg)	86 (17)	83 (20)
BMI (kg∙m^-2^)	28 (5)	28 (6)
Shoulder pain intensity during the last week (scale 0–10)	5.7 (2.0)	5.6 (2.2)
Elbow/Forearm pain intensity during the last week (scale 0–10)	4.2 (2.4)	4.1 (2.9)
Hand/Wrist pain intensity during the last week (scale 0–10)	3.7 (2.6)	3.9 (2.8)

Values are means (SD) * denotes significant difference between groups, P < 0.05.

### Interventions

The study aimed to implement two contrasting intervention modalities at the workplace. Lowering the physical exposure through ergonomic intervention is the traditional strategy to reduce the workload, i.e. this can be considered ‘usual care’. Conversely, an alternative strategy could be to increase individual’s physical capacity by means of strength training for the shoulder-, arm- and hand-muscles.

Participants were allocated to a 10 week intervention period and paralleled assigned to receive either strength training or participatory ergonomics at the worksite. The training group performed strength training of the shoulder, arm and hand muscles for 3 × 10 minutes a week, whereas subjects in the participatory ergonomic group received counseling on workstation adjustment and optimal use of work tools. These two ongoing interventions are described in detail in the following.

### Strength training intervention

Subjects randomized to this group (n = 33) performed supervised high-intensity specific strength training locally for the shoulder, arm and hand muscles. We prioritized a training program design that was cost-efficient and involved easy-to-use exercises and training equipment based on the assumption that subsequent post-intervention implementation at the current as well as other worksites will only be possible if the program is easily adopted, transparent and inexpensive to perform.

**Figure 2:**
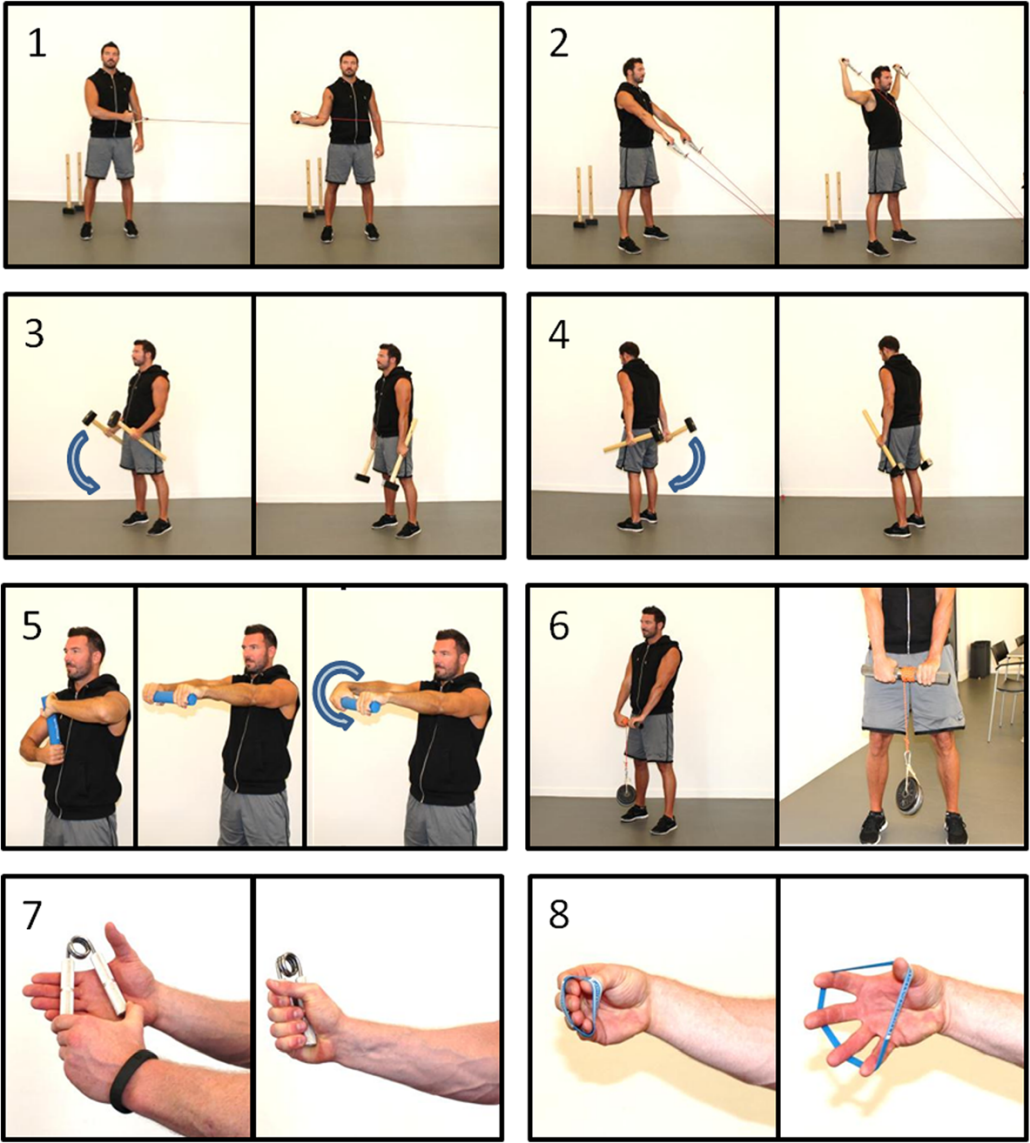
Exercises used in the strength training program: 1–2) shoulder rotation in two planes with elastic tubing, 3–4) ulnar and radial deviation of the wrist using sledgehammers, 5) eccentric training of the wrist extensors using a flexbar (Tyler twist), 6) wrist flexion and extension using a wrist roller, 7) flexion of the hand using a hand gripper, 8) extension of the hand using hand bands.

The training program consisted of 8 resistance exercises: (1–2) shoulder rotation in two planes with elastic tubing (Thera-Band), (3–4) ulnar and radial deviation of the wrist using sledgehammers, (5) eccentric training of the wrist extensors using a FlexBar (Thera-Band), (6) wrist flexion and extension by the use of one wicked wrist roller (IronMind), (7) flexion of the hand using a Captains of Crush gripper (IronMind), (8) extension of the hand using expand-your-hand bands (IronMind). All exercises are illustrated in Figure 2.

#### Training progression

Training intensity (loads) was progressively increased throughout the 10 week intervention period according to the principle of periodization and progressive overload (23). The exercises, shoulder rotation, ulnar and radial deviation and flexion and extension of the hand, were performed in a conventional manner using consecutive concentric and eccentric muscle contractions in a controlled manner. For these exercises relative loadings were progressively increased from 20 repetitions maximum (RM) at the beginning of the training period to 8 RM during the later phase. The Tyler twist performed with a FlexBar was only performed eccentrically. The wrist roller exercises were always placed at the end of the training session and only performed until full exhaustion using a load of 20–30 RM. 3–4 of the 8 different exercises with 3 sets per exercise were performed during each training session in an alternating manner.

All training sessions took place in designated training rooms located at the worksites. All sessions were supervised by a training instructor, who instructed the participants to correctly perform the exercises, and helped with exercise adjustment when needed. The instructors focused on positive feedback and social engagement to maintain motivation throughout the intervention period. At the first training session each participant received exercise equipment for hometraining (red and green Thera-Band elastic tubing and a green Thera-Band Hand Xtrainer) in case of absence from work (e.g. vacation).

#### Exercise adjustment

In case of acute worsening of pain or other contraindications during the time of training, the instructor used the following 4-stage model to subsequently adjust the specific exercise.

Stage 1: Reduce loading intensity. A reduction in load (kg lifted or resistance of elastic tubing) was implemented in the specific exercise that caused an increase in acute pain in the shoulder, arm, wrist or hand. A load reduction of up to 100% can be necessary, i.e. performing the movement without external resistance.

Stage 2: Reduced movement velocity. If a reduction in load fails to address the problem the movement velocity should be reduced.

Stage 3: Reduced range of motion (ROM). As a final action to solve the problem, the ROM should be reduced to the point where pain is not worsened. However, it is important not to decrease ROM too much since a reasonable part of dynamic strength training is desired interventional wise.

Stage 4: Interrupt the exercise. If none of the above stages is solving the problem, the specific exercise should be abandoned.

### Participatory ergonomic intervention

Subjects randomized to this group (n = 33) participated in ergonomic training and counseling, which is a typical worksite approach for the prevention and/or treatment of musculoskeletal disorders. The rationale for this intervention is to ensure that employees are sufficiently informed about the ergonomic hazards to which they may be exposed to and thus are able to participate actively in their own protection. The overall purpose is treatment and prevention of musculoskeletal disorders by avoiding excessive physical loads throughout the working day by handling each job task in the most appropriate ergonomically way.

#### Worksite analysis

Professional and experienced ergonomists with existing knowledge about ergonomic risk factors on the specific slaughterhouses provided information necessary to identify ergonomic hazards in the workplace. Based on the information about specific workstations that puts the employees at risk of developing musculoskeletal disorders, or other work-related disorders, a specially trained “ergonomic group” on each slaughterhouse executed a job hazard analysis for each task so identified. The specific work-station analysis focused on tasks that involve frequent and repetitive work. The analysis identified all risk factors present in each studied workstation and included: (1) measurements of repetitiveness (manipulations per cycle, cycle time and the total manipulations or cycles per work shift) (2) force evaluations (light, moderate or heavy), (3) tools, personal protective equipment, dimensions and adjustability, (4) shoulder, arm and hand postures and movements were assessed for the level of risk, (5) lifting hazards.

#### Hazard prevention

Based on the identification of ergonomic hazards through the worksite analysis the ergonomic group, in correspondence with professional ergonomists, developed a system for hazard prevention and control. A top priority focus for the two companies involved in this investigation was to design an intervention program for proper and safe work techniques that would be understood and adhered by managers, supervisors and workers. This program included: (1) sufficient and correct cutting techniques and work methods to improve body posture and reduce stress and strain on extremities, especially the arm, shoulder and wrist regions, (2) proper lifting techniques, (3) correct use of ergonomically designed work stations and fixtures, with focus on assembly lines and other stations that involves frequently and repetitive movement, (4) proficient knife care, including steeling and sharpening on a regular basis.

#### Ergonomic training and education

The participants in the ergonomic group received ergonomic training and education based on the practical outcomes of the hazard prevention system. The employees received ergonomic training during the intervention period with the majority placed during the initial weeks, which corresponds to the standard worksite ergonomic prescription. Further, educated supervisors associated to each department on the slaughterhouse monitored and helped employee to continue using proper work practice during the rest of the intervention period. The ergonomic training and counseling were implemented by professional ergonomists at the two slaughterhouses.

The majority of the ergonomic training was intended to address job specific hands-on training where participants received appropriate guidance in each individual work station. This training involved (1) use of proper cutting and lifting techniques, (2) care, use and handling techniques for knives and other relevant task-specific tools and devices, (3) use of appropriate guards and safety equipment.

### Blinding

Due to the interventional trial design, participants and instructors (i.e. strength training and ergonomics instructors) could not be blinded to group allocation. However, outcome assessors and data analysts were blinded to group allocation.

### Outcome measures

Outcomes were measured by trained clinical examiners and by questionnaire survey at baseline and after the 10 week intervention period.

#### Primary outcome measures

The primary outcome is the change from baseline to 10-week follow-up in pain intensity during the last week (average of 3 regions; shoulder, elbow/forearm and hand/wrist, respectively). Pain intensity was rated subjectively using a 0–10 modified VAS scale, where 0 indicates “no pain at all” and 10 indicate “worst pain imaginable” [36,40]. The shoulder, elbow/forearm and hand/wrist regions were defined by drawings from the Nordic questionnaire [41].

#### Secondary outcome measures

The work module of the Disability of the Arm, Shoulder and Hand questionnaire (DASH) was recorded and analyzed in the same way as the primary outcome. The participants replied to the following questions: “Select which best describes your physical ability in the past week. Did you have any difficulty.....1) Using your usual technique for your work? 2) Doing your usual work because of arm, shoulder or hand pain? 3) Doing your work as well as you would like? 4) Spending you usual amount of time doing your work?”. The possible answers to each question are: “No difficulty, Mild difficulty, Moderate difficulty, Severe difficulty, Unable” [19].

A physiotherapist performed a thorough clinical examination of the shoulder, arm and hand of all participants at baseline and follow-up physical examination, respectively. Examiner-verified palpable tenderness (scale ‘none’, ‘light’, ‘moderate’ and ‘severe’) of the muscles in the shoulder, arm and hand were summed up to a total tenderness score [36,42]. The change in the total tenderness score from baseline to week 10 was calculated.

Maximal muscle strength and function of the arm and hand was assessed by maximal isometric voluntary contractions in a custom-built dynamometer setting. The strength tests were part of the physical examination at baseline and follow-up.

### Sample size

A priori power analysis based on previous measurements revealed that 27 participants of each group for 95% power, SD of 1.5 and a minimal relevant difference of pain intensity of 1.5 [43] was sufficient to test the null-hypothesis of equality (α = 0.05). At an estimated 10% drop-out during the intervention period, group sizes were calculated to be at least 30.

### Statistical analysis

All statistical analyses will be performed using the SAS statistical software for Windows (SAS Institute, Cary, NC). The change in pain (0–10 scale) will be evaluated using a repeated-measures two-way analysis of variance (ANOVA) with *group*, *time* and *group by time* as independent variables. Subject is entered as a random effect. Analyses are adjusted for gender, workplace, age and pain intensity at baseline. We will perform all statistical analyses in accordance with the intention-to-treat principle using a Mixed model approach which inherently accounts for missing values. An alpha level of 0.05 will be accepted as significant. Outcomes will be reported as between-group least square mean differences and 95% confidence intervals from baseline to follow-up.

## Discussion

We will investigate the change in self rated pain intensity in the arm, shoulder and hand and the associated work disability of the participants in the two intervention groups. Ergonomic training aiming at reducing physical exposure and thereby workload is considered the standard prescription/conventional approach on prevention and treatment of musculoskeletal disorders in various worksites. However, increasing employee physical capacity by means of progressive strength training at the work site may represent an alternative way of reducing chronic pain and work disability in slaughterhouse workers. The present study will provide documentation to better guide workplace initiatives to reduce musculoskeletal pain among employees with repetitive and forceful work of the arms, shoulders and hands.

## Competing interests

The authors of the article declare that they have no conflict of interest what so ever. Further, the research has not received any funding or grant from any commercial source.

## Authors’ contributions

ES and LLA conceived the idea and design of the project and all authors participated in the methodologically development. ES, MDJ, CHA and KJ performed the clinical examination. All authors approved and critically reviewed the final version of the manuscript.

## Pre-publication history

The pre-publication history for this paper can be accessed here:

http://www.biomedcentral.com/1471-2474/14/67/prepub
